# The Rabies Free Burkina Faso initiative: an example of how one health-oriented civil society organizations can contribute towards the achievement of the rabies zero by 30 goal

**DOI:** 10.1186/s42522-023-00086-1

**Published:** 2023-07-21

**Authors:** Madi Savadogo, Laibané Dieudonné Dahourou, Abdoul Kader Ilboudo, Sidwatta Guy Ilboudo, Hamidou Zangré, Grissoum Tarnagda, Zacharia Souli, Alima Hadjia Banyala Combari, Ramata Diarra, Mémouna Bidima, Marina Gracienne Bintou Traoré, Charles Dieudonné Mandé, Kongnimissom Apoline Sondo, Katinka de Balogh

**Affiliations:** 1 Rabies Free Burkina Faso, Ouagadougou, Burkina Faso; 2 Institut de Recherche en Sciences de la Santé, Centre Nationale de la Recherche Scientifique et Technologique, Ouagadougou, Burkina Faso; 3 Fundamental and Applied Research for Animals and Health (FARAH), Faculty of Veterinary Medicine, University of Liege, Liege, Belgium; 4 Institut des Sciences de l’Environnement et du Développement Rural, Université de Dédougou, Dédougou, Burkina Faso; 5 International Livestock Research Institute, Ouagadougou, Burkina Faso; 6 Direction Générale des Services Vétérinaires, Ministère de l’Agriculture des Ressources Animales et Halieutiques, Ouagadougou, Burkina Faso; 7 Institut de l’Environnement et de la Recherche Agricole, Centre Nationale de la Recherche Scientifique et Technologique, Ouagadougou, Burkina Faso; 8 Unité de Formation et de Recherche en Sciences de la Santé, Université Joseph Ki-Zerbo, Ouagadougou, Burkina Faso; 9 Division of Infectious Diseases and Immunology, Department Biomolecular Health Sciences, Faculty of Veterinary Medicine, University of Utrecht, Utrecht, the Netherlands

**Keywords:** Public Health, Zoonoses, One health, Not-for-profit organization, Rabies education, Transdisciplinary

## Abstract

While technologies, tools and expertise have proven that countries can be made safe from dog-mediated human rabies, the disease remains a major public health threat in Burkina Faso. The paper reports the experience and success stories of Rabies Free Burkina Faso, an initiative established in 2020 as an example of civil society organization that promotes One Health for integrated rabies control in Africa. As recommended in the Global strategic plan, rabies elimination requires a systematic One Health approach, enhancing pre-exposure and postexposure prophylaxis, dog population management, dog vaccination, awareness raising, diagnosis, surveillance, funding as well as policies and regulations. Rabies Free Burkina Faso was established on 28 September 2020 as not-for-profit organization and aims to strengthen the use of a One Health approach as a non-governmental, multidisciplinary initiative dedicated to promoting rabies elimination. Categories of interventions developed by Rabies Free Burkina Faso cover awareness raising, training and One Health capacity building, dog rabies vaccination, seeking vaccines and providing support, including financial resource to communities to ensure that bite victims are appropriately provided with post-exposure prophylaxis, research, community engagement and joint outbreak investigation in collaboration with competent authorities. Reported success stories confirm the relevance of roles that can be played by Rabies Free Burkina Faso supporting animal health and human health authorities in the fields of rabies control and One Health development in the country.

## Background

Rabies is a serious challenge for animal health and human health, particularly in low- and middle-income countries like Burkina Faso. It affects wild and domestic animals, and is transmitted to humans predominantly through bites, licks and scratches from rabid animals, mostly dogs [1]. Despite some initiatives regarding rabies prevention and control having been implemented (dog vaccination, policy development, postexposure prophylaxis, awareness creation for general public or specific groups) by national authorities in charge of animal health and human health, the available data indicates that the disease has been present in Burkina Faso for decades [2–4]. Moving ahead, the global rabies elimination strategy developed by the Tripartite (World Health Organization, Food and Agriculture Organization of the United Nations, World Organisation for Animal Health) and Global Alliance for Rabies Control (GARC), recommends One Health action at national, regional and international levels to meet the global objective of zero human deaths from dog-mediated rabies by 2030 [5]. The One Health concept promotes multisectoral, transdisciplinary, transboundary, and community-oriented collaboration [6]. Global health threats such as emerging antimicrobial resistance, foodborne diseases, zoonoses (e.g. highly pathogenic avian influenza, tuberculosis, Ebola, rabies), and the COVID-19 pandemic highlighted that human health, animal health and environment health are interconnected [7, 8]. Overall, the importance of the One Health approach for rabies control is widely recognized, and the disease is even mentioned as a model for One Health capacity building [5]. According to the GARC website the theme identified for the celebration of World Rabies Day 2023 is «Rabies: All for 1, One Health for all» (https://rabiesalliance.org/world-rabies-day).

In Burkina Faso, canine rabies is endemic with several human cases recorded every year [2, 3, 9]. It is under-reported and the available data do not allow to determine the real burden of the disease in the country. The fight against rabies in the country involves different public and private entities, especially in human health and animalhealth sectors [10–12]. Postexposure prophylaxis is provided by two rabies vaccination centers located in Ouagadougou and Bobo Dioulasso, the biggest cities of the country. Health facilities across the country are involved in the management of human cases and surveillance of human rabies is performed by technical services of the Ministry of Health both at the national and local levels. When it comes to the prevention and control of animal rabies, interventions such as policy development, dog vaccination, laboratory diagnosis, and surveillance are conducted by the veterinary services of the Ministry of Livestock. In addition, private veterinarians play key roles, including dog vaccination, awareness raising, biting dog observation and rabies vaccine supply. A study conducted by Savadogo et al. provides a more in-depth characterization of actors and roles in rabies control in the country [12]. In the absence of an operational integrated bite case management approach [13], the communication and data sharing between the stakeholders involved in the control of the disease are lacking, especially between medical and veterinary services. Multi-sectoral engagement and One Health collaboration, including community education are needed for effective rabies control. Currently, it still appears, that the fight against rabies remains a concern of only government entities, leaving aside the potential contributions that civil society organizations could strengthen national efforts including resource mobilization.

This paper presents the model and success story of the Rabies Free Burkina Faso initiative, the first One Health oriented civil society organization dedicated to rabies prevention and control in Burkina Faso. It set out to enable rabies control and working at the frontline in the fight against rabies. It to supports government and partners’ efforts by strengthening awareness raising, building One Health capacity and mobilizing diverse stakeholders across disciplines for integrated surveillance and response (e.g. joint investigation, dog vaccination, postexposure prophylaxis). In what follows, we describe the different steps from the idea to the establishment of Rabies Free Burkina Faso, the areas of intervention and corresponding success stories, the challenges identified, and finally the key lessons learned. This paper intends to be inspirational for other countries encountering similar conditions and challenges in the elimination of dog-transmitted rabies.

## The establishment of rabies free Burkina Faso

### Reasons for creating a civil society organization

The relevance of civil society activism for improved health systems has been documented in the Global South, for example in the fields of health policy making, acquired immunodeficiency syndrome control as well as maternal and reproductive health [14–16]. In response to the prioritization of rabies and a recognized need of strengthened control of the disease in the country, Rabies Free Burkina Faso was launched in 2020. It is an example of building on achievements of the rabies community, demonstrating concrete accomplishments in the fight against the disease. In order to better inform decision-makers, founding members of the association have devoted many years to conduct situational research and build evidence on the rabies epidemiological status [3, 9] as well as develop an understanding of community knowledge, attitudes and practices [17, 18]. Subsequently, during the national One Health zoonotic diseases prioritization (OH-ZDP) conducted in 2017, rabies was ranked among the top five priority zoonotic diseases together with anthrax, tuberculosis, highly pathogenic avian influenza, and dengue [19].

This One Health zoonotic disease prioritization enabled key stakeholders (Ministry of Livestock, Ministry of Health, Ministry of Environment and Wildlife) and partners to develop multisectoral collaboration and coordination mechanisms. Taking examples from other countries in the sub-region [20–22], Burkina Faso set up a national One Health coordination platform (Plateforme Nationale de Coordination One Health) in 2019 with the aim of providing stakeholders with a legal framework for the operationalization of the One Health approach [12]. Despite this government policy, this did not result in any significant initiatives for rabies control in the country. For example, the situation is characterized by low rabies surveillance, low dog rabies vaccination coverage, the lack of national rabies vaccination campaigns as well as frequent postexposure prophylaxis shortages. Moreover, following the outbreak of the SARS-CoV-2pandemic, in 2020 the annual World Rabies Day organized by the government as the national flagship to raise awareness, was cancelled. This was not surprising as rabies, despite its high burden in terms of human deaths in more than 150 endemic countries [23], is still considered a neglected infectious disease. The vicious circle of neglect of rabies described in African countries is very illustrative and has hindered the progress towards the elimination of the disease [24, 25].

Nevertheless, since high level political will is increasing as shown by the selection of rabies among the top five priority zoonotic diseases, and the establishment of a the national One Health coordination platform [26], founding members of Rabies Free Burkina Faso started thinking about mechanisms to address gaps and how best to support the government efforts through an action-oriented approach. Finally, on September 20, 2020, after the launch of the United Against Rabies Forum and the joint appeal by the Directors General of the Tripartite, the founding members decided to create a multidisciplinary organization dedicated to animal and human rabies elimination in Burkina Faso. This led to the establishment of Rabies Free Burkina Faso on 28 September 2020, highlighting the importance of World Rabies Day, as no government activity was planned to commemorate this global event that year.

### Setting-up rabies free Burkina Faso to operationalize one health

As required by national regulations concerning non-for-profit organizations, the process to establish Rabies Free Burkina Faso culminated in the official approval by the Ministry of Territorial Administration [27]. The approval is issued once the government is aware of the missions and areas of intervention of the entity to be created.

Phase one consisted of preparatory activities. All required legal documents (statutes, and rules of procedure) were elaborated according to the national requirement, and validated by a working group composed of veterinarians and medical doctors, as well as participants to be invited to a one day One Health workshop on 28 September 2020. Professionals from different disciplines (animal health, human health, environment, wildlife, anthropology and social sciences, communication and journalism, biology, statistics, economics and management) and sectors (public, private, NGOs) were invited to this meeting with the objective of discussing the roles of their respective disciplines in rabies control, and the establishment of multidisciplinary association : Rabies Free Burkina Faso for strengthened One Health action for rabies control in Burkina Faso.

The workshop took place in Ouagadougou, the capital city of Burkina Faso. During the first half day, three presentations on animal and human rabies were provided. The first speaker was from the animal health sector and presented the epidemiology and the management of animal rabies. The second presentation was provided by the human health sector on the occurrence of human rabies cases and prevailing gaps in rabies prevention in humans in Burkina Faso. Discussions following these presentations highlighted a lack of rabies awareness among the general public [9], limited access to postexposure prophylaxis for people exposed to rabies [28] and the need of capacity strengthening for animal health and human health students and professionals [12, 29]. Finally, the last speaker focused on the One Health concept and its added-value in achieving animal and human rabies elimination, in line with the Zero by 30 global objective [30]. The enthusiastic mobilization and discussions between the participants on this occasion demonstrated that such an integrated initiative was long needed by the actors.

**Fig. 1:**
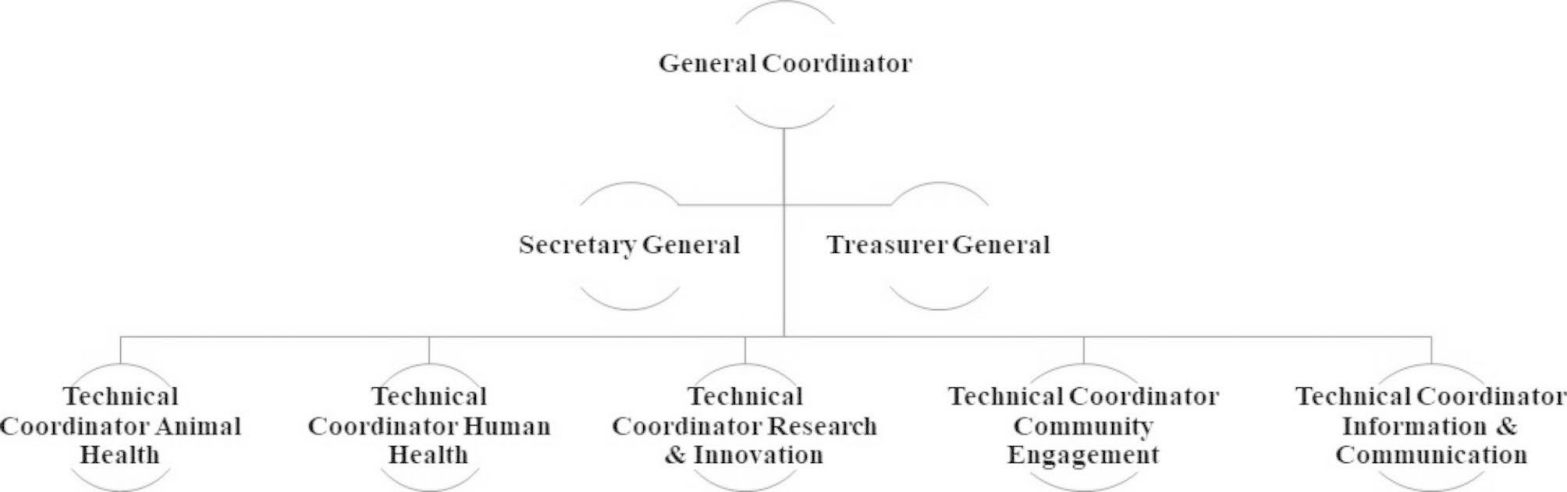
: Organization chart of Rabies Free Burkina Faso

On the second half day, all participants agreed to co-found a multidisciplinary association dedicated to tackle rabies and validated the legal documents that had been developed (statutes, and rules of procedure), authorizing the appointment of a Coordination Committee for Rabies Free Burkina Faso. Embracing the spirit of the One Health concept, five Technical Coordination groups were established under the Coordination Committee : Animal Health, Human Health, Community Engagement, Communication, and Research & Innovations (Fig. 1).

**Fig. 2:**
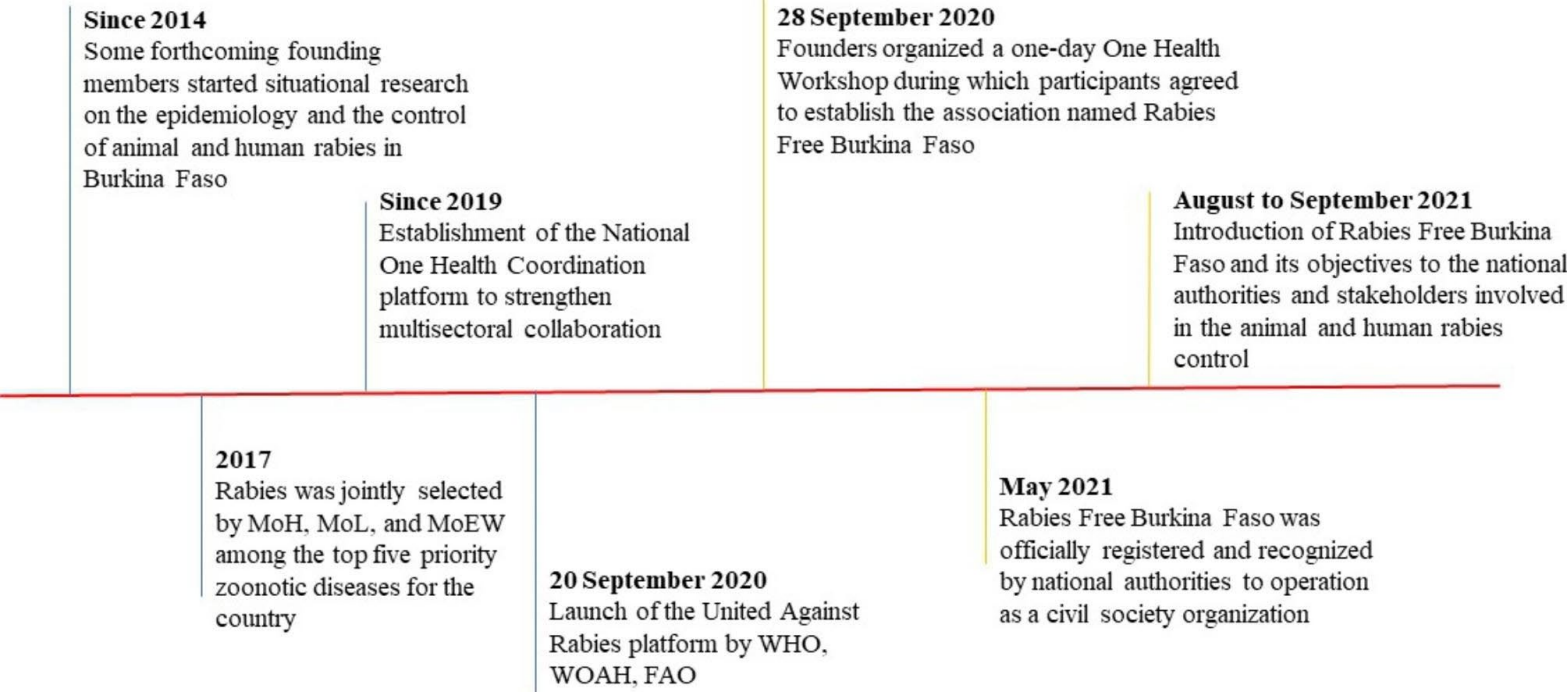
Timeline summarizing most important events and steps having characterized the process of creation of Rabies Free Burkina Faso. MoH : Ministry of Health ; MoL : Ministry of Livestock ; MoEW : Ministry of Environment and Wildlife ; WHO : World Health Organization ; WOAH : World Organisation for Animal Health ; FAO : Food and Agriculture Organization of the United States

The competent authorities were informed about the establishment of the civil society association. Subsequently, Rabies Free Burkina Faso was approved by the Ministry of Territorial Administration under registration number 000001115801 issued on 18 May 2021 (Fig. 2). In addition, the creation of the association was published in the Official Journal of Burkina Faso (N°37, page 3065–3066, dated 16 September 2021). Since then, Rabies Free Burkina Faso has become part of national associations permitted to operate in the field of rabies control and related public health issues.

## Areas of interventions and success stories

The most importante activities conducted by Rabies Free Burkina Faso since its establishment (September 2020) to date are presented in Table 1.

**Table 1:** Summary of key activities and contributions of Rabies Free Burkina Faso to animal and human rabies control in Burkina Faso

Categories of intervention	Activities hosted/participated	Participants / Targeted groups
Information, education and communication	September 28, 2020 : One day One Health workshop on animal and human rabies control in Burkina Faso in the framework of World Rabies Day celebration	Professionals in charge of animal health, human health, livestock, wildlife, as well as researchers and teacher-researchers
	March 21, 2021 : Hosting an interactive radio program sensitizing population on dog vaccination and postexposure prophylaxis	Dog owners, bitten persons, animal health professionals, human health professionals, general public
	September 12, 2021 : Hosting an interactive radio program on rabies prevention and control, and the role of Rabies Free Burkina Faso initiative	Dog owners, bitten persons, animal health professionals, human health professionals, general public
	September 25 & 26, 2021 : Hosting two awareness sessions with children about dog bite management and rabies prevention	School children, parents and supervisors
	September 28, 2021 : Hosting radio and TV programs disseminating key messages on rabies prevention and control in the framework of World Rabies Day celebration	Dog owners, bitten persons, animal health professionals, human health professionals, general public
	January 9, 2022 : One day awareness campaign (focus group and individual discussions) held in the Rural Commune of Sabou	Dog owners, household members
	July 21, 2022 : Online public conference on One Health and rabies control in Burkina Faso, in collaboration with Food and Agriculture Organization (FAO) and United Against Rabies Forum (UAR)	Animal health, human health, wildlife and related professionals from Burkina Faso and the West African sub-region
	On continual basis : developing and disseminating key messages related to rabies control using social media such as Facebook (https://www.facebook.com/Rabifreebf), Linkedin (https://www.linkedin.com/in/rabies-free-burkina-faso-b71910221/), Twitter (https://twitter.com/RabiesFreeBF), WhatsApp and Website (http://rabiesfreebf.org/ )	General public both at national and international levels
	From September 25 to October 6, 2022 : Continued TV and radio broadcasting to raise awareness in the framework of World Rabies Day and World Animal Day (Animal care and prevention of diseases in animal, rabies and rabies prevention, dog vaccination, responsable animal ownership and implications for human health and well-being)	General public both at national and international levels
Dog vaccination	September 31 to October 4, 2020 : Participating in a mass dog vaccination campaign organized by the Ministry of Livestock in the city of Ougadougou	Dog owners
	March 1 to 31, 2021 : Participating in mass a dog vaccination campagn organized by the Provincial Directorate of Livestock in Bobo Dioulasso	Dog owners
	January 9, 2022 : One day dog vaccination campaign (combining fixed point and door-to-door approaches) held in the Rural Commune of Sabou	Dog owners
	October 7 to 9, 2022 : Three days dog vaccination campaign (combining fixed point and door-to-door approaches) held in the area of Tengandogo located in Ouagadougou	Dog owners
Postexposure prophylaxis	November 8, 2020 : Providing advice and financial support to initiate and secure postexposure vaccination for a child bitten by a roaming dog suspect for rabies	Bitten persons, family members
	January 2, 2021 : Providing advice and financial support to initiate and secure postexposure vaccination for three children and one adult bitten by a confirmed rabid dog in Ouagadougou	Bitten persons, family members
	August 28, 2021 : Providing advice and financial support to initiate and secure postexposure vaccination for three children and one adult bitten by a roaming dog suspect for rabies	Bitten persons, family members
	August 30, 2021 : Providing advice and financial support to initiate and secure postexposure vaccination for one child bitten by a cat suspect for rabies	Bitten persons, family members
	September 6, 2021 : Providing advice and financial support to initiate and secure postexposure vaccination for two children bitten by a dog suspect for rabies	Bitten persons, family members
	March 23, 2022 : Providing advice and financial support to initiate and secure postexposure vaccination for three children bitten by a confirmed rabid dog in Ouagadougou	Bitten persons, family members
Surveillance	November, 2021 : Initiating investigation that allowed laboratory confirmation of rabies in a suspected community goat (reported to be bitten by a roaming dog) in the Commune of Sabou	High risk community members and their owned animals
	November, 2021 : Organisation of a joint investigation (involving animal health and human health sectors) in the community affected by a goat rabies case	Dog owners, breeders, local animal health professionals, local human health professionals, municipality officers, Regional directorates in charge of human health and animal health
Contribution to research-based evidence data collection	Different members of Rabies Free Burkina Faso were involved in research topics covering rabies epidemiology and surveillance [4, 11], dog vaccination [24, 31, 37], dog ownership [9, 31], postexposure prophylaxis [3, 9, 28, 29], One Health in rabies control [10, 12, 24], community awareness about rabies [9, 15, 18], implications of dog and dog meat trading on rabies risk and control in communities [35]	Community members, public and private entities involved in rabies control and prevention
Partnership building related activities	July 26 to 27, 2021 : Invited to attend the canine show (education on ownership and relevance of dog walking and vaccination for welfare) organised by Centrale Canine du Burkina Faso in Ouagadougou	Dog owners, general public
	September to October 2021 : Invited to share Rabies Free Burkina Faso experience during the United Against Rabies Forum Annual Meeting [4]	Global rabies leading experts, national authorities, international partners, animal and public health professionals
	February, 2022 : Invited to contributions to the national One Health strategic planning workshop hosted by the National One Health Coordination Platform in Koudougou, Burkina Faso	National stakeholders and partners involved in One Health operationalization process
	March 9 to 11, 2022 : Invited to contribution to the SARE assessment in the framework of the National Strategic Plan for rabies elimination	National stakeholders and partners involved in rabies prevention and control
	April 4 to 15, 2022 : Five members of Rabies Free Burkina Faso attended the International Training on Rabies Surveillance and control, held in Abibjan, Cote d’Ivoire. A presentation on the success stories of Rabies Free Burkina Faso initiative was given to participants [36]	Animal and human health professionals and young researchers trained on rabies on rabies control with a focus on One Health capacities development in West and Central African regions
	August, 2022 : Invited to contribution to the elaboration of National Zoonoses thematic committee regulatory documents hosted by the National One Health Coordination Platform in Ouagadougou, Burkina Faso	National stakeholders and partners involved in zoonoses control
	August 23 to 26, 2022 : Invited to contribute to the zoonotic risk joint assessment workshop hosted by the National One Health Coordination Platform in Ouagadougou, Burkina Faso	National stakeholders and partners involved in zoonoses control
	September 28, 2022 : Invited to talk about experience of Rabies Free Burkina Faso on the use of the One Health approach for rabies control	Global rabies leading experts, national authorities, international partners, animal and public health professionals

### Education and awareness raising

Rabies Free Burkina Faso engaged in education and awareness raising among community members, students and professionals (animal health, human health, environment, etc.) regarding rabies prevention and control, especially since several studies evidence a lack of knowledge among the general public regarding animal species mostly involved in rabies transmission, routes of transmission, importance of dog vaccination, management of biting dogs, and postexposure prophylaxis including washing of bite wounds [3, 9, 31]. Addressing these gaps, Rabies Free Burkina Faso used social media, such as Facebook, Twitter, LinkedIn and WhatsApp, to disseminate key messages on rabies prevention and control. In addition, members of the association provided radio and TV interviews to a range of online media, published articles on the disease control situation in the country, organized training workshops for professionals involved in the control of rabies, and presented oral communications during school events as well as national seminars and conferences (Table 1). As reported by various studies, up to 45% of human rabies cases occurred in children less than 15 years old [3, 23, 32]. Therefore, during World Rabies Day celebrations in 2021, awareness sessions were conducted within two ‘’amusement parks for children’’ located in Ouagadougou. These sessions aimed at sensitizing children about animals involved in rabies transmission, appropriate behaviours regarding roaming dogs, how to act in the event of being bitten by an animal, and the importance of notifying bites to their parents or other adults. Accordingly, flyers with key messages targetted children, their parents or accompanying adults were distributed. Some feedbacks collected by the team of Rabies Free Burkina Faso from the parents of attending children at the end of the awareness sessions confirmed the relevance of such community outreach.

*« We are very grateful, we will spread messages received to stop rabies in our country »* (A mother attending a session).

*« These are simple measures. I heard about rabies when I was in primary school. Therefore, it’s a good initiative to remind us what to do to avoid rabies.* » (A father attending a session).

### Improving access to postexposure prophylaxis for exposed persons

The two only post-exposure prophylaxis centers in Burkina Faso (located in the two biggest cities : Bobo Dioulasso and Ouagadougou) register nearly 10,000 bites cases each year (unpublished post-exposure prophylaxis facility records). Exposed persons have difficulties in accessing post-exposure prophylaxis, especially in remote areas. Direct and indirect costs associated with post-exposure prophylaxis are prohibitive for the general population and even worse, frequent post-exposure prophylaxis shortages increase the risk of persons bitten to contract rabies [33, 34]. In such situations, Rabies Free Burkina Faso uses its network and social media channels to source post-exposure prophylaxis from private pharmacies from across the country, and at times from further afield. However, vaccine sourced from private pharmacies is at least 10 times more expensive than vaccines administered in a government post-exposure prophylaxis centers, making it difficult for many families to afford these costs. In spite of limited financial resources, Rabies Free Burkina Faso was able to secure complete post-exposure prophylaxis for 13 vulnerable bite victims between January 2021 and March 2022 in Ouagadougou. However, if nothing is done to improve the availability, the accessibility and the delivery of rabies vaccines and immunoglobulins, any communication and outreach effort can lead to frustrations, loss of trust in health facilities, and in the long run become counterproductive.

### Joint outbreak investigation and response

**Fig. 3:**
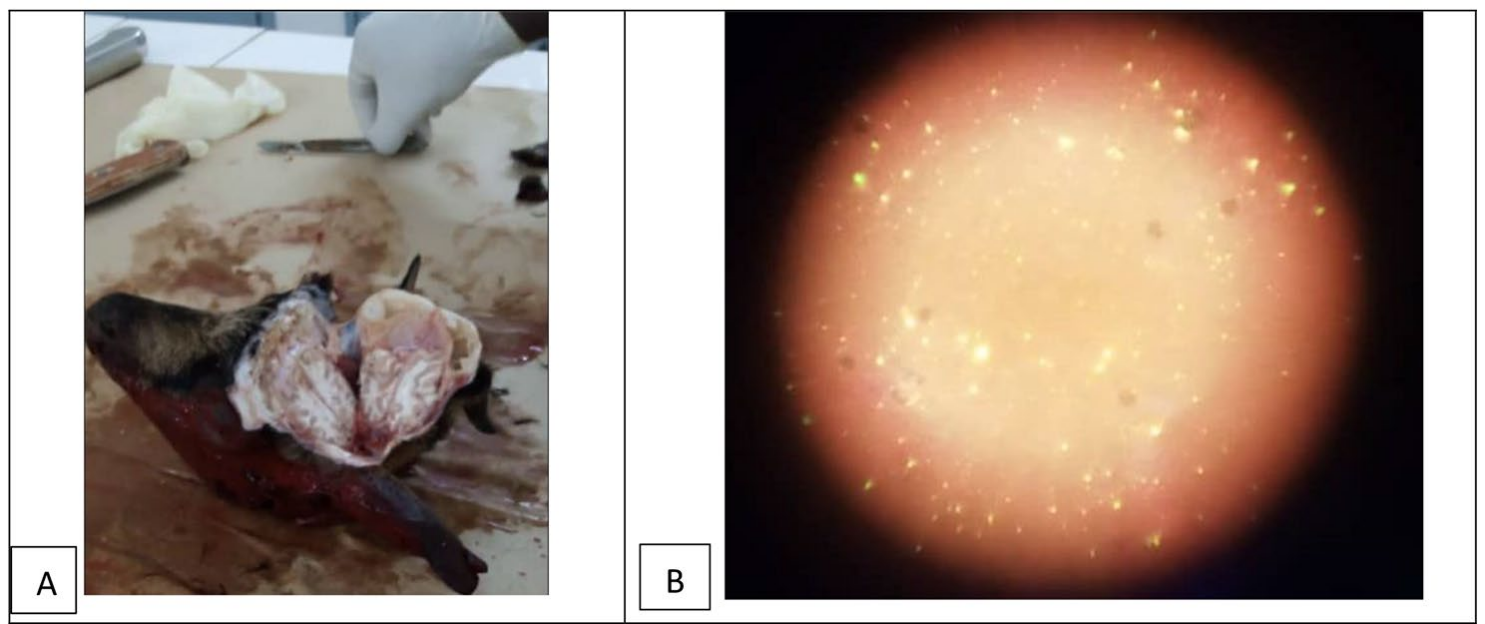
Suspected rabid goat sample collection (**A**) and reading of the performed Fluorescent Antibody Test (**B**)

As reported in Tanzania [13], joint outbreak investigation is a major component of the integrated bite case management approach and key for the improvement of rabies surveillance and postexposure prophylaxis. Whenever Rabies Free Burkina Faso has been alerted on a bite case, if possible, a field visit is conducted to discuss with the local community, the local authorities as well as veterinary and medical officers, to search for unreported bite cases and provide appropriate guidance to community members. For example, a suspected case of goat rabies was reported in November 2021 in Sabou, located 80 km from Ouagadougou. To the best of our knowledge, the authorities did not take any action by themselves. Therefore, the suspected goat was removed from its owner by the association, placed under veterinary observation by local animal health workers, and during the first week it died and a brain sample was submitted for laboratory investigations (Fig. 3A). As the laboratory results confirmed rabies in the goat (Fig. 3B), the association conducted an outbreak investigation and response activities on site. This rabies case in a goat implies that, although around 98% of human rabies cases in the African countries are caused by dogs, the possibility of rabies virus transmission by livestock should not be overlooked in awareness messages, especially for agropastoral and rural communities. In Addition, local animal ownership practices being characterized by free-roaming of dogs and livestock, rabies cases in livestock are probably caused by infected dogs. An outbreak investigation team composed of veterinary and medical professionals conducted one day interviews with local decision-makers, animal health and human health workers, as well as the goat owner and local community members. Discussions with the stakeholders included the search for further information on the outbreak (e.g. possible contacts occurred between the rabid goat and humans or other animals), briefing the local animal health workers and human health workers on bites and rabies cases notification and management, sensitizing the livestock owning households on rabies, and identifying through a participatory approach the response actions to be taken. Indeed, one of the main recommendations that emerged from the discussions with the stakeholders was the organization of a campaign to vaccinate carnivores and sensitize communities. Therefore, following the investigation, Rabies Free Burkina Faso, in collaboration with local stakeholders (animal health and human health authorities, animal health office, human health center, municipality, private veterinarians, community animal health workers, and community leaders), organized a mass dog rabies vaccination campaign on 9 January 2022. Fixed point and door-to-door vaccination were conducted by multidisciplinary teams (each including one animal health worker and one human health worker) resulting in the vaccination of owned dogs (69) and cat (01) and senitization of over 140 community members to the appropriate practices in terms of dog ownership, the importance of dog vaccination and postexposure prophylaxis for rabies prevention and control. To the best of our knowledge, this is the very first time that such a joint rabies outbreak investigation followed by the implementation of disease control interventions (dog vaccination and community sensitization) was conducted in Burkina Faso. In addition, the success recorded throughout the response to this outbreak demontrates the relevance of adequate communication and cooperation between sectoral stakeholders involved in a community-oriented health intervention.

### Advancing research on rabies

Rabies Free Burkina Faso is also committed to increase engagement of decision-makers and different stakeholders (public animal health and human health entities, non-government organisations, private enterprises, communities) through advocacy. As the disease has been long neglected [34], there was a lack of reliable data to inform prevention and control policies. This gap highlights why Rabies Free Burkina Faso has such an important role to play. Therefore, understanding the importance of research-based evidence in advocacy and communication, the association contributed to generating data on various aspects related to rabies control. Different members of the association contributed to studies conducted in the country on topics that covered rabies epidemiology, risk associated with dog meat trade, dog vaccination coverage, dog ownership practices and challenges as well as opportunities for One Health collaboration for more effective rabies control (Table 1). Through the thesis supervision for final year-students in animal health and human health studies [35], our research activities provide an excellent channel creating interest and thus engaging the future professionals with the challenges of rabies control.

## Main challenges identified

Although significant results have been achieved, these first two years of action have still encountered some challenges, such as :

- Lack of awareness about rabies – Both the general public and a significant proportion of professionals (human and animal health sectors) in contact with communities do not have adequate knowledge about rabies prevention and control measures. This lack of knowledge about rabies and its associated socio-economic as well as public health burden remains a major obstacle to stakeholders’ commitment.
- Lack of resources – Even though the disease is now considered as a national priority zoonosis, entities involved in rabies control are chronically underfunded, including Rabies Free Burkina Faso. In the specific case of Burkina Faso, this situation results in inadequate dog vaccination coverage and frequent postexposure prophylaxis shortages.
- Absence of rabies strategic plan – A national integrated strategic plan is currently being developed and needs to ensure synergic and well-coordinated interventions among stakeholders, including civil society associations like Rabies Free Burkina Faso.

## Key lessons learned

Beside the challenges experienced, operating as a multidisciplinary, frontline association allowed the emergence of several lessons, pinpointed as follow :

- The contribution of civil society associations to public entities is critically important for increased health promotion within communities. Indeed, as an association, Rabies Free Burkina Faso offers a practical framework free from bureaucracy that characterizes many public agencies. As several members of the association are working for government or private agencies (e.g. Ministry of Health, Ministry of Livestock, Ministry of Environment, Universities, Research Institutes, Private clinics, Media stations), it further enables the mobilization of local expertise for cost-effective and highly impactful interventions. However, a close collaboration with public agencies, including Ministry of Health, Ministry of Livestock and the National One Health Platform, remains critical for sustainable actions.
- In addition to conventional media channels (radio and TV), mobile technologies, internet and popular social media (Facebook, LinkedIn, Twitter, WhatsApp) are innovative channels for information and communication dissemination among key stakeholders (including young population, professionals, decision makers, communities) both at country and global levels. At several times, Rabies Free Burkina Faso used social media for sourcing postexposure prophylaxis in private pharmacies across the country.

## Conclusion and way forward

Beside the government efforts, enhancing initiatives towards increased health promotion, such as prevention of dog-mediated human rabies within the general public, requires the involvement of all relevant individuals and agencies. The Rabies Free Burkina Faso initiative constitutes a platform that puts together professionals (from a wide range of disciplines and backgrounds) and non-professionals, increasing commitment to support the government efforts towards rabies elimination. In addition, given its multidisciplinary character, Rabies Free Burkina Faso offers a concrete example of promoting One Health for integrated rabies control at a non-government level. Therefore, the platform plans to further develop strong institutional grounding and an extended network through regional, provincial and local committees to further strengthen rabies prevention and control in Burkina Faso.

The lessons learned from setting up such an association allows us to make a few recommendations. Indeed, in a socio-political context characterized by an increasing lack of confidence in civil society initiatives, paying attention to a certain number of points in the approach may facilitate compliance of the stakeholders to such a multi-disciplinary platform.

- **Engagement and expertise**: The leaders of the initiative must be credible, with a strong knowledge of and commitment to rabies prevention and control;
- **Awareness**: The stakeholders need to be sensitized on the benefits of the One Health approach, particularly in the current context of increased emerging zoonoses (e.g. rabies) and other public health issues;
- **Relevance**: The leaders of the initiative should be convinced that rabies is a real health problem and that each person’s knowledge and skills are required to make rabies history by achieving the Zero By 2030 goal.

## Data Availability

Not Applicable.

## Abreviations

GARC: Global Alliance for Rabies Control
OH-ZDP: One Health Zoonotic Diseases Prioritization
NGOs: Non Government Organizations
